# Targeting the innate immune system in pediatric and adult AML

**DOI:** 10.1038/s41375-024-02217-7

**Published:** 2024-03-08

**Authors:** Alicia Perzolli, Joost B. Koedijk, C. Michel Zwaan, Olaf Heidenreich

**Affiliations:** 1 Princess Máxima Center for Pediatric Oncology, 3584 CS Utrecht, The Netherlands; 2 Department of Pediatric Oncology, Erasmus MC/Sophia Children’s Hospital, 3015 GD Rotterdam, The Netherlands; 3 Wolfson Childhood Cancer Research Centre, Newcastle University, Newcastle upon Tyne, NE1 7RU UK

**Keywords:** Acute myeloid leukaemia, Innate immunity

## Abstract

While the introduction of T cell-based immunotherapies has improved outcomes in many cancer types, the development of immunotherapies for both adult and pediatric AML has been relatively slow and limited. In addition to the need to identify suitable target antigens, a better understanding of the immunosuppressive tumor microenvironment is necessary for the design of novel immunotherapy approaches. To date, most immune characterization studies in AML have focused on T cells, while innate immune lineages such as monocytes, granulocytes and natural killer (NK) cells, received less attention. In solid cancers, studies have shown that innate immune cells, such as macrophages, myeloid-derived suppressor cells and neutrophils are highly plastic and may differentiate into immunosuppressive cells depending on signals received in their microenvironment, while NK cells appear to be functionally impaired. Hence, an in-depth characterization of the innate immune compartment in the TME is urgently needed to guide the development of immunotherapeutic interventions for AML. In this review, we summarize the current knowledge on the innate immune compartment in AML, and we discuss how targeting its components may enhance T cell-based- and other immunotherapeutic approaches.

## Introduction

Acute myeloid leukemia (AML) is a heterogeneous hematological malignancy characterized by uncontrolled clonal proliferation of myeloid progenitor cells in the bone marrow [1, 2]. Although survival has improved considerably over the past decades, about 70% of adult AML and 20–30% of pediatric AML patients in high income countries do not survive due to treatment-related toxicity and disease relapse [1–3]. Moreover, survivors often suffer from serious treatment-related side effects and long-term impaired quality of life [2]. Hence, targeted therapies are urgently needed to improve outcomes and to reduce treatment-related toxicity.

The success of several traditional AML therapies such as allogeneic hematopoietic stem cell transplantation (allo-HSCT) and donor lymphocyte infusion relies on the ability of T and NK cells to recognize and eliminate leukemic cells, and illustrates the sensitivity of AML to functional immune cell cytotoxicity [4–6]. Although these observations support the use of immunotherapy for the treatment of AML, these traditional therapies are often accompanied by major side effects such as graft-versus-host disease, severe infections, organ toxicity and infertility [7, 8]. Interestingly, innovative T cell-based immunotherapies such as bispecific antibodies and chimeric antigen receptor (CAR) T cells hold the promise of immune-mediated tumor eradication with fewer side effects, as shown in the treatment of B-cell precursor ALL (BCP-ALL) with blinatumomab and CAR T cells [9]. Unfortunately, clinical trials of T cell-based immunotherapies in adult AML have shown disappointing efficacy, while trials for pediatric AML have only recently been initiated (e.g., NCT03825367) [3, 10, 11].

The challenges in translating the successes of these novel immunotherapies to AML may be explained by several factors, including genetic and clonal heterogeneity, paucity of suitable leukemic targets and the immunosuppressive tumor microenvironment (TME). For AML, the alleged TME is the bone marrow (BM) and includes, next to tumor cells, immune and other normal hematopoietic, stromal, and endothelial cells [12]. Studies in both adult and pediatric AML patients have shown that the BM of a subgroup of patients is immune-depleted (or “cold”), which has been associated with reduced responses to T cell-based immunotherapies in both AML and solid cancers [13–17]. To date, most studies that investigated the TME of AML focused on T cells, while less attention has been paid to the innate immune compartment [11, 18, 19]. However, there is ample evidence that innate immune cells, such as tumor-associated macrophages (TAMs), neutrophils, and myeloid-derived suppressor cells (MDSCs), contribute to a suppressive TME [20, 21]. Furthermore, they are at the basis of immunotherapy failure in both hematological and solid malignancies [20, 21]. For example, a study in human lung cancer showed that TAMs are an important determinant for the development of a T cell-excluded tumor phenotype and that depletion of TAMs improved the efficacy of anti-PD1 immunotherapy [22]. Also in adult AML, studies have shown that macrophages become avidly pro-tumorigenic in the TME [23, 24] and that their abundance is associated with non-response to the bispecific antibody flotetuzumab (CD3xCD123) [13]. Thus, a better understanding of the innate immune compartment in the TME of AML will be critical for the design of successful immunotherapeutic approaches for this disease.

In this review, we summarize the current knowledge on the innate immune system in adult and pediatric AML and highlight how these cells interact with AML blasts in the BM microenvironment. Moreover, we discuss how innate immune cells may be harnessed to improve immunotherapy efficacy and ultimately, the outcome of AML patients.

## The innate immune system in the tumor microenvironment of AML

**Fig. 1:**
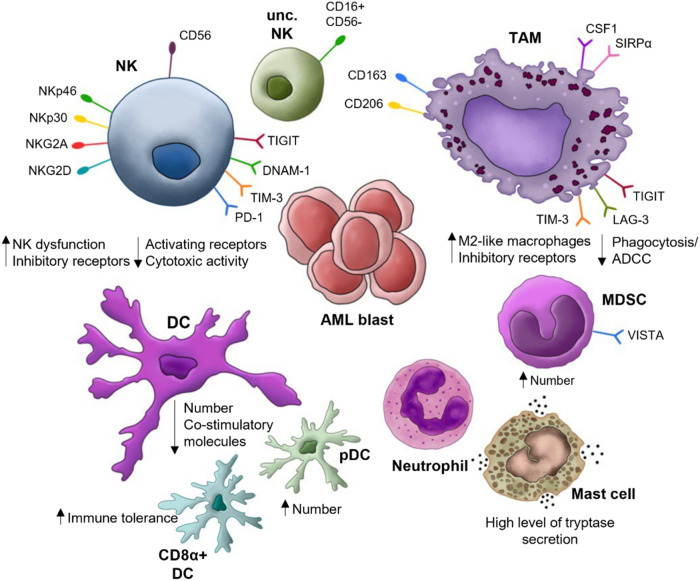
Overview of relevant innate immune cell populations in the tumor microenvironment in AML. NK Natural killer cell, unc. NK unconventional natural killer cell, TAM tumor-associated macrophage, MDSC myeloid-derived-suppressor cell, Mast cell, Neutrophil, DC dendritic cell, pDC plasmacytoid dendritic cells, CD8α^+^ DC CD8α^+^ dendritic cell, AML blast.

AML development and progression are associated with dysregulated immune responses and induction of an immunosuppressive TME [11, 18, 19]. In both solid and hematological malignancies, innate immune infiltrate in the TME determines at least in part the cancer progression and the cancer’s sensitivity to standard chemotherapies, targeted therapies, and immunotherapies [25–28]. Furthermore, AML blasts can hide from immune recognition by modifying the number and the phenotype of adaptive and innate immune cells in the TME. Aside from promoting T cell exhaustion and expansion of T regulatory cells (Tregs), AML blasts have been found to increase the number of MDSCs, polarize macrophages toward a pro-tumoral phenotype, and hamper NK cell effector functions [26, 29–31]. Moreover, AML cells can dysregulate the innate immune response by releasing cytokines and soluble factors or through direct contact with innate immune cells [32]. In the following sections, we will summarize the knowledge about the phenotype and the role of the innate immune cells in the TME and how they interact with AML blasts (Fig. 1).

### Tumor-associated macrophages

Macrophages are critical cellular components of the immunosuppressive TME. The standard classification divides macrophages into pro-inflammatory M1-like macrophages and anti-inflammatory M2-like macrophages [33]. In infected tissues, macrophages are first polarized to an M1-like phenotype to assist the host against pathogens. Subsequently, macrophages are polarized to an M2-like phenotype to form an anti-inflammatory response, as the breaks to stop inflammation and to repair damaged tissue [34]. In the context of cancer, the convention of breaking down macrophages into two highly polarized states is now being replaced with the concept of high plasticity and susceptibility of macrophages’ phenotype and function to signals derived from the TME [35, 36]. In many cancer types, macrophages are driven to an M2-like functional program, which are also referred to as tumor-associated macrophages (TAMs). TAMs have been associated with tumor progression, tissue remodeling, and poor prognosis [21, 37, 38].

In adult AML, multiple studies have reported increased frequencies of M2-like macrophages in the BM in comparison to healthy donors, while M1-like macrophages were less abundant [39, 40]. Furthermore, single-cell RNA-sequencing of end-of-induction BM in pediatric AML revealed a predominance of M1-like macrophages in patients that remained in complete remission after standard chemotherapy, while enrichment of M2-like macrophages was identified in patients that relapsed [41]. In addition, we previously identified that a predominance of M2-like macrophages was associated with non-response to the bispecific T cell-engager flotetuzumab [13]. Thus, it appears that M2-like macrophages play an important role in supporting leukemogenesis and therapy resistance, while M1-like macrophages may have anti-tumoral properties in AML.

**Fig. 2:**
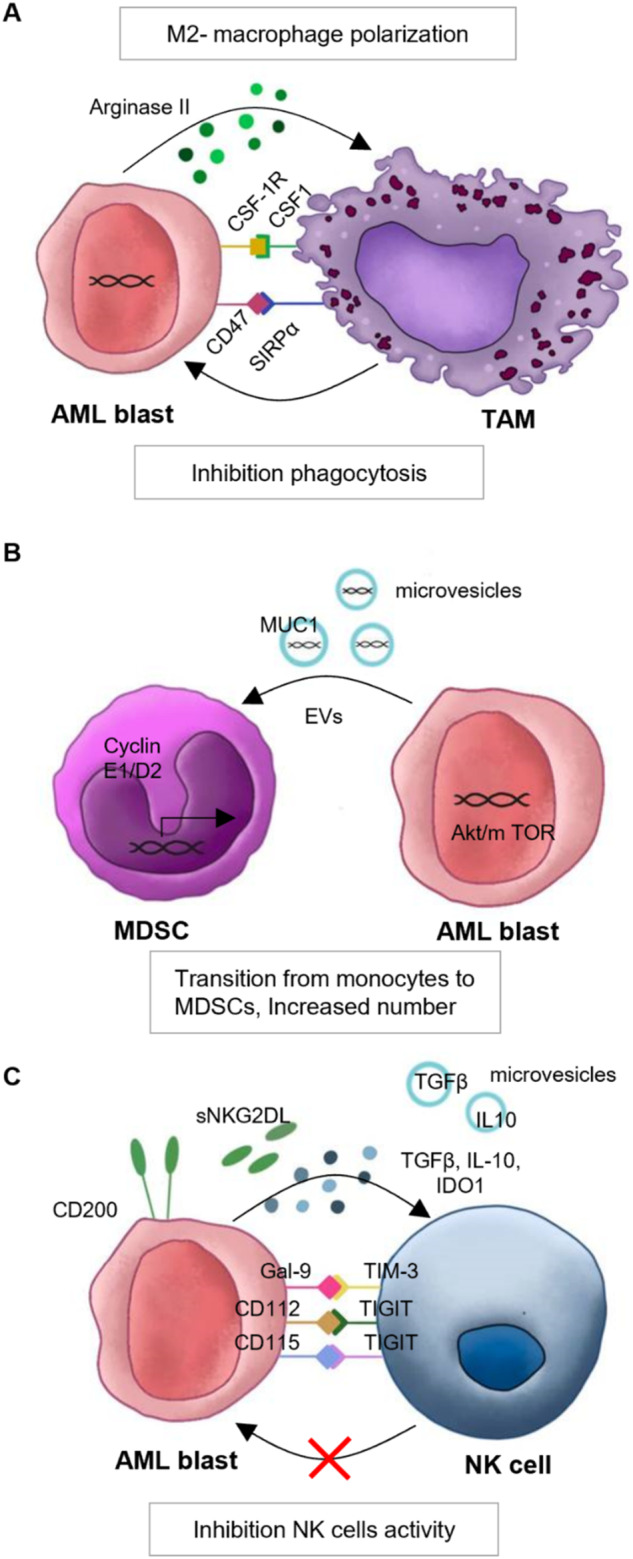
Overview of cross-talks between AML blasts and innate immune cells in the TME. **A** AML blasts polarize macrophages toward an M2-like phenotype (through arginase II secretion and via CSF1R). AML blasts escape phagocytosis by macrophages through the expression of CD47. **B** AML blasts secrete extracellular vesicles (EVs), containing the protein MUC1, leading to MDSCs expansion. **C** AML blasts inhibit NK cell cytotoxicity by activating immune checkpoints and secreting several factors, such as TGFβ, IL-10, IDO1, sNKG2D and microvesicles containing TGFβ, IL-10.

AML blasts can directly drive macrophages toward an M2-like phenotype by the production of immunosuppressive enzymes or by the activation of transcription factors (Fig. 2A). For example, the release of arginase II by AML cells has been shown to induce an M2-like phenotype and simultaneously, to inhibit T cell proliferation [42]. Alternatively, AML cells ability to polarize macrophages to an M2-like phenotype may be mediated by the transcription factor Growth Factor Independence 1 (Gfi1) [24]. In various AML mouse models, the absence of Gfi1 (in blastocytes) could reprogram macrophages associated with AML and polarize them toward an antitumor state both in vitro and in vivo. Indeed, the co-culture of Gfi1-WT BM-derived macrophages with AML cell lines significantly increased the expression of M2-like surface markers. Contrarily, BM-derived macrophages derived from Gfi1 KO mice were enriched for M1-like macrophages [24].

Furthermore, to evade immune surveillance, AML blasts aberrantly express ligands for “do not eat me” signals, such as the integrin-associated protein CD47 (Fig. 2A). CD47, by binding to its receptor signal regulatory protein-alpha (SIRPα) on anti-tumor macrophages, promotes AML cell survival by blocking macrophage phagocytosis [43]. An anti-CD47 monoclonal antibody has been shown to induce antileukemic phagocytosis in preclinical and clinical studies [44–46]. Therapies targeting the CD47/ SIRPα axis are already in clinical trials for adult AML, which we discuss in the second part of this review. Overall, these data suggest that AML blasts can induce phenotypic switching of macrophages toward a leukemia-supporting phenotype, suggesting that macrophages represent an interesting therapeutic target.

In addition, macrophages can remodel the TME through the interaction with immune cells and corresponding changes in their number, activity and phenotype. From solid cancers, it is known that macrophages in the TME are one of the main regulators of T cell infiltration into the tumor [47, 48]. Specifically, M1-like macrophages are known to be the primary source of T cell-attracting chemokines, while M2-like macrophages suppress T cell proliferation and cytotoxicity by, e.g., the depletion of L-arginine, and recruit Tregs by, e.g., the secretion of CCL22 [47, 48]. In our laboratory, we found that M2-like macrophage predominance negatively correlated with T cell infiltration in the BM of pediatric AML, suggesting a role for macrophages in regulating T cell infiltration in the BM [13]. Further investigations, such as dynamic imaging microscopy are needed to understand how macrophages may control the distribution and migration of T cells in the TME of AML, as this may provide insights on how macrophages could be leveraged to enhance immunotherapy responses.

Altogether, the available evidence suggests a complex interaction between AML blasts and M2-like macrophages. It will be crucial to understand how this interaction contributes to the immunosuppressive TME, immune evasion, tumor progression and the suppression of T cell immunity. Investigating the molecular mechanisms driving this process may facilitate the development of potential therapies focused on reverting M2-like macrophage to an M1-like macrophage state, thereby (partially) restoring anti-tumor immunity of the TME.

### Dendritic cells

In contrast to M2-like macrophages, a high density of dendritic cells (DCs) at the tumor site may be beneficial because of their ability to present extracellular antigens on major histocompatibility complex (MHC-I) class I molecules to enable antitumor CD8^+^ T cell activation [49]. Two subsets of DCs with distinct functions have been identified: conventional DCs (cDCs) and non-conventional DCs, which include plasmacytoid DCs (pDCs) [49]. cDCs develop from myeloid progenitor cells, while pDCs are generated via both myeloid and lymphoid precursors [50]. In solid tumors, distinct DC subsets have been associated with different outcomes [17]. For instance, a high density of cDCs has been associated with tumor regression, a high abundance of tumor-infiltrating lymphocytes (TILs), increased T cell activation and better overall survival (OS) in several solid tumors [51, 52]. In contrast, pDCs are thought to contribute to establishing an immunosuppressive TME by inducing Tregs activation through IL-10 or IDO secretion [53]. Moreover, in primary breast cancer, pDCs have been shown to correlate with an unfavorable prognosis [54].

In adult AML, cDCs are diminished or even absent in the BM of AML patients compared to healthy donors, which may contribute to the lack of CD8^+^ T cells in the TME [55]. Nevertheless, a recent single-cell RNA-sequencing study demonstrated a critical role for cDCs in the immune recognition of AML. It has been found that antigens from circulating leukemic cells are primarily captured and cross-presented by a subset of splenic cDCs, named CD8α^+^ DCs, mediating leukemia antigen recognition by antigen-specific CD8^+^ T cells in vivo [56]. These data suggest that AML immune tolerance may be initiated at the level of the innate immune system. In contrast to a low level of cDCs, a high number of pDCs has been found in AML patients [57]. Furthermore, high levels of pDCs are negatively associated with OS after allo-HSCT in adult AML [58]. Taken together, the lack of cDCs and high levels of pDCs are likely to contribute to the establishment of an immunosuppressive niche in AML.

### Neutrophils

Neutrophils are polymorphonuclear phagocytes and despite their cytotoxic capacity, they are now recognized to promote immunosuppression, tumor growth, and metastasis in solid cancers [59]. Also, an increased number of neutrophils in the peripheral blood (PB) has been associated with a poor response to immune checkpoint inhibitors (ICIs) in renal cancer patients [60]. Neutrophils engaged in the TME are called tumor-associated neutrophils (TANs). In most human tumors, with few exceptions, increased levels of TANs have been associated with poor prognosis [61].

Most data characterizing neutrophils is derived from the analysis of solid tumors, whereas few studies have investigated this in hematological malignancies. An analysis of the pediatric AML TARGET cohort revealed an association between the abundance of neutrophils and poor prognosis [62]. Furthermore, in adult AML, a high neutrophil-to-lymphocyte ratio has been associated with poor prognosis and linked to adverse clinical outcomes, including lower response rates to standard treatment, shorter OS, and higher relapse rates [63, 64]. Altogether, the role of neutrophils in AML remains incompletely understood. Uncovering their contribution to the immunosuppressive TME could provide tools to tailor current immunotherapy strategies and pave the way for myeloid cell-centered therapeutic strategies.

### Mast cells

Mast cells are crucial cells participating in both innate and adaptive immune processes that play important roles in the maintenance of many physiological functions as well as in the pathophysiology of diseases [65]. Mast cells are commonly seen in various tumors and have been associated with both tumor rejection and tumor promotion [66]. After recruitment to the TME by various chemokines, mast cells are activated [66] and release a variety of cytokines and growth factors [67].

In several tumors, such as colorectal cancer and follicular lymphoma, low numbers of tumor-infiltrating mast cells predicted prolonged OS [68, 69], and in melanoma, tumor-infiltrating mast cells have been shown to co-localize with Tregs and have also been associated with resistance to anti-PD1 therapy [70]. In AML, little is known about the presence and function of mast cells in the BM microenvironment. However, peripheral blood from AML patients showed a higher level of tryptases and serine proteases that are abundantly expressed by mast cells compared to healthy controls [71]. Further investigations about mast cell number and their interaction with AML blasts are needed to understand whether mast cells may contribute to the immunosuppressive niche in AML patients and tumor growth.

### Myeloid-derived suppressor cells

Myeloid-derived suppressor cells (MDSCs) are a heterogeneous group of immature myeloid cells (both monocytic and granulocytic), with potent immunosuppressive activity [31]. For instance, MDSCs may differentiate toward suppressive macrophages or neutrophils, inhibit NK cell-mediated tumor cell lysis, recruit Tregs, and prevent T cell proliferation and activation, in both solid and hematological malignancies [72–74].

In adult AML, an expansion in the number of MDSCs has been reported in PB and BM aspirates [31] and high levels of MDSCs correlated with worse outcomes [75, 76]. However, the specific processes that drive MDSC expansion and how they contribute to the immunosuppressive microenvironment in AML are incompletely understood. Preclinical studies have shown that MDSCs in murine models suppress T-cell responses and T-cell proliferation and that the expansion of MDSCs is driven by the secretion of extracellular vesicles (EVs) secreted by AML cells [31] (Fig. 2B). These EVs are absorbed by myeloid progenitor cells and induce their expansion by MUC-1-mediated expression of c-myc (Fig. 2B) [31]. Interestingly, upregulation of MYC and downregulation of its negative regulator miR-34a have also been associated with the increased expression of PDL-1 in AML patients with TP53 mutations [77]. Furthermore, a recent study reported that AML-derived EVs play a key role in the differentiation of monocytes into MDSCs [78]. Preclinical studies suggested that palmitoylated proteins on the surface of these EVs are required for MDSC differentiation via activation of the Akt/mTOR pathway [78]. Inhibiting protein palmitoylation could therefore be a potential option to interfere with MDSC differentiation. In support of this mechanism, several studies in solid tumors have shown the secretion of EVs containing microRNAs and proteins that have a significant impact on tumor proliferation and immune suppression [79, 80].

Finally, MDSCs can also drive AML immune-escape mechanisms. For instance, high expression of the immune regulatory VISTA has been found on MDSCs from AML patients, which inhibited T cell responses [81]. Significant efforts are currently being undertaken to therapeutically interfere with the immunosuppressive function of MDSCs to boost the efficacy of ICIs or other therapies [82, 83].

### NK cells

NK cells are innate lymphoid cells with potent cytotoxic activity in anti-cancer immune responses. In patients and animal models, a deficiency of NK cells has been associated with an increased incidence of various cancer types, illustrating their role in immune surveillance [84]. NK cells can recognize malignant cells with absent MHC class I expression, a common feature in pediatric tumors [85, 86], followed by the elimination of tumor target cells through the release of cytotoxic granules that contain perforin and granzymes [87]. NK cell activities are tightly regulated by the balance between activating and inhibitory receptors. In both solid and hematological malignancies, NK cells display a similar profile as seen in exhausted T cells, characterized by up-regulation of inhibitory receptors, decreased proliferative capacity, and reduced expression of IFNγ, granzyme B, TNFα, and impaired antibody dependent cell-mediated cytotoxicity via CD16 [88, 89].

Clinical studies observed a significant reduction in the number of NK cells in AML patients compared to normal values and the frequency of NK cells was inversely proportionate with prognosis and OS [90, 91]. Furthermore, studies found that NK cells of AML patients have a significantly lower capacity to secrete IFNγ and showed numerous signs of an exhausted phenotype, as compared to healthy controls [92, 93]. These include underexpression of activating receptors such as NKG2D, DNAM-1 and NKp30 as well as overexpression of inhibitory receptors such as NKG2A and KIR2DL1 [93, 94].

In addition to NK cell abnormalities, AML blasts themselves exhibit modified expression of ligands for NK cell receptors (Fig. 2C). As a result, the effectiveness of NK cell-mediated responses against AML blasts is diminished. For instance, AML blasts overexpress the ligands CD137 and CD200, whose interaction with their respective receptors on the surface of NK cells leads to the suppression of NK cell cytotoxicity and IFNγ production [34, 95]. Moreover, AML blasts showed decreased expression of the NK cell activating ligand NKG2DL and the ability to release a soluble form of NKG2DL causing the downregulation of NKG2D receptors on NK cells and impairing their cytotoxic activity [32, 33]. In contrast, high expression levels of the inhibitory molecules galectin-9, CD112, and CD115 were found on AML blasts, which might decrease NK cell activation [96, 97]. Furthermore, AML blasts can also decrease NK cell cytotoxicity by releasing microvesicles containing TGFβ and IL-10 [98, 99] or by the secretion of cytokines such as TGFβ and IDO1, which also correlate with elevated Tregs number and suppression of T cell proliferation [100, 101] (Fig. 2C).

Lastly, immune checkpoints (ICs) on NK cells play an important role in NK cell activation and cytotoxicity [102]. For instance, a study demonstrated increased PD-1 (programmed cell death 1), TIGIT (T cell immunoreceptor with Ig and ITIM domain) and TIM-3 (T-cell immunoglobulin and mucin domain-3) expressions on NK cells from AML patients at initial diagnosis compared to NK cells from healthy donors [103]. In addition, the study showed that TIGIT^+^ NK cells exhibited a lower antileukemic effect compared to TIGIT^-^ NK cells, and AML patients with a relatively high frequency of TIGIT^+^ NK cells had a worse prognosis [103]. Antibodies targeting PD-1 and TIM-3 are currently being explored in multiple clinical trials for adult AML patients (e.g., NCT02397720, NCT03940352).

Overall, there is ample evidence that NK cells in AML are functionally impaired and are characterized by unconventional phenotypes and defective maturation. NK cell-based immunotherapies are of considerable interest in this disease. We will highlight below strategies for manipulating NK cells in activating the reconstitution of NK cells against AML.

## Immunotherapies targeting the innate immune system in AML

Over the last few decades, considerable progress has been achieved in the treatment of AML with the intensification of chemotherapy, risk-adapted treatment, improvements in supportive care and allo-HSCT and the development of new drugs (e.g., gemtuzumab ozogamicin and *FLT3*- and menin inhibitors). However, refractory and relapsed disease remains a major issue [104].

In both solid tumors and hematological malignancies, long-lasting clinical responses have been achieved with T cell-based immunotherapies [105, 106]. Unfortunately, the efficacy of these immunotherapies in adult AML appears to be limited and relatively unexplored in its initial stages in pediatric AML [11]. Problems include the need for allo-HSCT due to prolonged myelosuppression, harvesting issues, rapid disease progression, infectious complications and organ toxicity.

As discussed in previous chapters, innate immune cells in the TME are critical determinants of resistance to T cell-based immunotherapies limiting the infiltration of cytotoxic T cells and recruiting Tregs. Therefore, targeting innate immune components may help to overcome the immunosuppressive TME and to increase the effectiveness of cancer therapy. Alternatively, NK cell-based immunotherapy represents a relatively novel immunotherapeutic strategy, unleashing immune suppression of NK cells to attack various cancers [107]. Indeed, NK cells are considered promising alternatives to modified T cells, with more favorable toxicity profiles and relatively low manufacturing costs [108]. However, the application of NK cell-based immunotherapies for AML is still at the initial stage, especially for pediatric AML [107, 108].

Below, we discuss several immunotherapeutic strategies based on innate immune cells, which may promote the effect of T/NK-cell engaging immunotherapies to ultimately improve the outcome of AML patients.

### Targeting myeloid cells in AML

Preclinical studies have provided ample evidence that myeloid cells influence the efficacy of about every type of cancer therapy, ranging from chemotherapy, radiotherapy, targeted therapy to immunotherapy [109–113]. Over the years, an increasing number of clinical studies has been published on targeting myeloid cells in cancer. In general, approaches to target myeloid cells with tumor-promoting characteristics can be divided into two main strategies: (1) reducing their number by depleting them or by blocking their recruitment, and (2) repolarizing them toward an anti-tumor phenotype (Table 1). In this way, the functionality of anti-tumor effector immune cells, such as NK cells and CD8^+^ T cells, may be restored in both solid and hematological malignancies, as well as their infiltration into malignant tissues.

**Table 1:** Overview of strategies to target myeloid cells in AML.

Strategies	Targets
Deplete myeloid cells	• Targeting CCL2/CCR2 and M-CSF/M-CSFR pathways to inhibit monocytes recruitment to the tumor site and TAM maintenance; • Blocking CXCL8 or CXCR1 and CXCR2 to inhibit neutrophils mobilization and recruitment; • Interfering with VEGF-VEGFR signaling, contributing to TIMs migration and differentiation.
Reprogram myeloid cells into antitumor effectors	• Triggering TLRs: increased immunogenicity (DCs and NKs activation, conversion from M2-like to M1-like macrophages); • Cytokines: IL-12 associated with reprogramming of MDSCs, DCs, and TAMs; • Manipulation of transcription factors: STAT3, NF-kB and PI3Kγ; • Inhibition of enzymes expressed by TIMs (IDO-1, ARG-1, iNOS).
Targeting cell surface receptors	• “Don’t eat me signals”: CD47/SIRPα, CD24/SIGLEC10 • Activating receptors: CD40/CD40L • Immune checkpoint inhibitors on macrophages

#### Manipulating myeloid cell numbers

Different strategies including pharmacological and genetic approaches have depleted myeloid cells from the TME, successfully controlling tumor progression in various solid tumor-mouse models [114, 115]. These strategies mainly target pathways involved in the recruitment/trafficking of myeloid cells in the TME. The most well-studied chemokine and cytokine pathways that have been implicated in the recruitment of suppressive monocytes and macrophages to the TME in solid tumors include the CCL2-CCR2 axis, the CCL5-CCR5 axis and the CSF-CSF1R axis [116]. A study demonstrated that targeting the CCL2-CCR2 axis may be a promising approach in AML as well [113]. Indeed, the blockade of the CCL2-CCR2 axis interfered with the infiltration of M2-like macrophages, especially in the spleen of mice engrafted with AML. Alternatively, targeting the mobilization and recruitment of neutrophils, by blocking CXCL8 or the chemokine receptors CXCR1 and CXCR2 [117] is now being evaluated in patients with advanced solid tumors [118].

Preclinical studies in both adult and pediatric AML applying these strategies are not available yet.

#### Reprogramming myeloid cell phenotypes/activity

An alternative strategy to counteract the immunosuppressive TME is to shift the balance of anti- and pro-leukemic activities in favor of the former. This includes the manipulation of genetic programs and epigenetic mechanisms that dictate the phenotype and the function of myeloid cells.

Triggering Toll-like receptors (TLRs) on myeloid cells has been extensively studied in this context. TLRs are activated by bacterial and viral products and their activation promotes inflammatory gene expression (via NF-kB and IFNs pathways) [119]. TLR agonists have been developed as a therapy for solid tumors to induce macrophage repolarization and stimulate a potent proinflammatory polarization in myeloid cells. The most common agonists that control myeloid cell phenotypes are ligands binding TLR3, 4 and 7–9. For example, in solid tumors, it has been found that TLR9 agonists, e.g., CpG oligodeoxynucleotide DNA, increased the number of tumor-infiltrating lymphocytes in the TME, while MDSCs, TAMs, and Treg cells were reduced [120, 121]. TRL9 agonists are now in clinical trials for melanoma and colorectal cancer (NCT03618641, NCT03507699). This field is less investigated in hematological malignancies. Nevertheless, several results demonstrated that TLR signaling increased the immunogenicity of AML cells as well, making them more vulnerable to T cell-mediated invasion [122, 123]. For example, a study reported that TLR7/8 agonists could induce DC activation and IL-2 production consequently activating NK cells in AML patients [123].

Cytokines represent another way of functionally reprogramming myeloid cells. Tumor-induced IL-10 was found to be specifically responsible for DC-dysfunction in response to antigen-driven maturation. It has been reported to block the differentiation of monocytes into DCs and to promote their maturation into macrophages in AML [124, 125]. Higher plasma concentrations of IL-10 were observed in AML patients not responding to the treatment in comparison with complete remission patients [126]. Contrarily, IL-12 has been shown to reduce new vessel formation, induce apoptosis, and inhibit tumor cell proliferation in AML [127]. Delivery of IL-12 in TME has been associated with reprogramming of MDSCs, DCs, and TAMs into antigens presenting with CD8^+^ T cell activity capacity [128, 129].

Alternatively, direct manipulation of transcriptional factors such as STAT3 and NF-kB, or kinases such as PI3Kγ may be promising strategies to reverse the activity of immunosuppressive myeloid components in the TME [130, 131]. In a pre-clinical study of a mouse model that mimics inv(16) AML in humans, systemic administration of STAT3 siRNA resulted in the eradication of established AML in mice. Interestingly, STAT3 inhibition failed to prevent leukemia progression in immunodeficient mice, demonstrating that the anti-leukemia effects of STAT3 inhibition were likely immune-dependent [132, 133]. Ablating STAT3 through inhibitors or knock-out results in enhanced function of DCs, T cells, NK cells, and neutrophils, and induced phenotype switching from M2 to M1-like macrophages in tumor-bearing mice [134, 135].

Lastly, inhibition of enzymes expressed by tumor-infiltrated myeloid cells, such as IDO-1, ARG-1, or iNOS, might reverse the TME and enhance T cell activity. Inhibitors for these enzymes were found to block the suppressive activity of MDSCs and enhance the number and function of tumor-specific T cells [136, 137]. Studies in pediatric AML have shown a negative correlation of IDO-1 mRNA expression with outcomes, supporting the idea that these patients can potentially benefit from specific IDO-1 inhibitor therapies [138].

Overall, strategies that modulate rather than ablate myeloid cells may not only harness their antitumor properties but also reverse the immunosuppressive phenotype of the TME. Therefore, it will be important to study whether clinical interventions that manipulate myeloid cells have therapeutic benefits in AML patients and whether myeloid cell-targeting agents mitigate the limitations of other treatments.

#### Targeting cell surface receptors on myeloid cells in AML

In more recent years, specific surface receptors on tumor myeloid-infiltrating cells have been targeted with antibodies. As noted previously, tumor cells evade phagocytosis by overexpressing “don’t eat me signals”, such as CD47 and CD24, which interact with their binding partners SIRPα and SIGLEC10, respectively, that are abundantly expressed on innate immune cells [139, 140]. The phagocytosis of tumor cells and the presentation of tumor antigens to T cells by antigen-presenting cells, such as macrophages and DCs, are essential for effective T cell-mediated antitumor responses [141]. In AML, CD47 expression predicted worse OS in three independent cohorts of adult AML and was found to be more highly expressed in AML leukemic stem cells than their normal HSPCs [45]. Clinical studies confirmed the importance of inhibiting CD47/SIRP-α interaction and several CD47/SIRP-α mAbs or fusion proteins are currently in phase I/II, as a single or combination therapy for adult AML (NCT05266274, NCT04980885, NCT02641002, NCT02678338, NCT04755244, NCT05263271, NCT05607199, NCT05367401, NCT04214249). For example, the anti-CD47 antibody magrolimab induces tumor phagocytosis and eliminates leukemic stem cells [45]. Unfortunately, the clinical study Phase 3 (Enhance 3, NCT05079230) evaluating the safety and the efficacy of magrolimab versus placebo in combination with azacytidine in newly diagnosed AML patients, has been recently stopped due to futility. Antibodies or fusion proteins targeting CD47 are not in clinical trials in pediatric AML yet.

Like the regulation of T and NK cells by activating and inhibitory checkpoint receptors, the differentiation of myeloid cells into immunostimulatory and immunosuppressive phenotypes is governed by various regulatory molecules. For example, the CD40:CD40 ligand interaction tilts the myeloid immune cell population in favor of an anti-tumor instead of an immunosuppressive phenotype, and this is accompanied by an increased level of activated T cells in the tumor tissue, suggesting this interaction as a promising target [124, 142–144]. The administration of an agonistic anti-CD40 antibody resulted in enhanced anti-AML T cell immunity and prolonged survival in a murine AML model [143]. Moreover, the expression of inhibitory checkpoints such as TIGIT, TIM3 and LAG3 have been found on macrophages in AML patients. Interestingly, these three inhibitory receptors were mainly expressed on M2-like macrophages [144]. Previous studies showed in vivo models that overexpression of TIGIT and TIM3 induce M2-like polarization [145, 146]. Hence, blocking TIGIT, TIM3 and LAG3 may be a strategy to repolarize M2-like macrophages toward the M1-like phenotype.

Overall, several monoclonal antibodies targeting myeloid checkpoints, e.g., CD47, PD-1, LILRB, TREM2, have been recently under development and clinical trials are ongoing for solid tumors [147]. Immunotherapeutic strategies targeting myeloid cells are still limited in adult AML and absent in pediatric AML, except for therapies targeting CD47 and NK cells.

### Targeting pediatric AML via NK cells

The graft-versus-leukemia effect observed after allo-HSCT in AML patients indicates the susceptibility of leukemic cells not only to T cells but also to NK cell cytotoxicity [148]. NK cell alloreactivity is triggered by the mismatch between killer-cell immunoglobulin-like receptors (KIRs) on donor NK cells and human leukocyte antigen (HLA) class I molecules on recipient cells [148]. Ongoing studies emphasize the efficacy of NK cell-based immunotherapies to reconstitute NK cell cytotoxicity against AML, to induce and maintain remission in adult AML patients. These therapies include adoptive NK cell transfer, CAR-NK cells, antibodies, cytokines and immunomodulatory drugs. A recent review has summarized the current knowledge of different NK cell-based immunotherapies in adult AML, and we refer to that article for a broader overview of this topic [107]. Here we focus on NK cell-based immunotherapies in pediatric AML, which are still very limited (Table 2).

**Table 2:** Overview of NK cell-based immunotherapies in clinical trials for pediatric AML (R/R: relapse/refractory).

Therapy	Disease stage	Age (in years)	Phase	Conditioning therapy	Year	NCT
NK cell transplant (haploidentical)	R/R AML	<30	2	Chemotherapy (Busulfan, cyclophosphamide)	2007–2020	NCT00553202
	R/R AML	<120	2	Chemotherapy (Cyclophosphamide, fludarabine)	2007–2016	NCT00526292
	BM transplantation within the past 4–6 weeks	<65	1	–	2007–2013	NCT00569283
	R/R AML	>2	2	Chemotherapy (Cyclophosphamide, fludarabine)	2006–2017	NCT00274846 [159]
	R/R AML	<18	2	Chemotherapy (Cyclophosphamide, fludarabine)	2009–2010	NCT00871689
	R/R AML	4–35	1	Stem cell infusion	2011–2019	NCT01287104 [160]
Umbilical cord blood transplant	R/R AML	<18	2	Chemotherapy (Cyclophosphamide, fludarabine), Total-body irradiation	2009–2012	NCT00871689
	R/R AML	<45	2	Chemotherapy, Radiation	2006–2012	NCT00354172
Memory-like NK	R/R AML	1–21	2	Chemotherapy (Fludarabine, Ara-C)	2020–ongoing	NCT04354025
	R/R AML	>1	2	CD3+ T Cell Product Infusion	2017–ongoing	NCT03068819 [151]
	R/R AML	>2	2	Chemotherapy (Cyclophosphamide, fludarabine), IL-2	2013–2023	NCT01898793 [161]
Anti-CD38 and FT538 (target therapy)	R/R AML	>11	1	Chemotherapy (Cyclophosphamide, fludarabine)	2021–ongoing	NCT04714372
TriKe (CD163-NKp46-CD16a)	R/R AML	>1	1 2	–	2021–ongoing	NCT05086315
JK500 cell injection	R/R AML	<18	Early 1		2022–ongoing	NCT05519384

In line with the data obtained in adult patients, pediatric AML may also be successfully targeted with alloreactive NK cells [149]. Clinical trials in children with AML mainly involve the infusion of haploidentical NK cells or donor umbilical cord blood NK cells. Adoptive therapy using haploidentical NK cells has been shown to be safe in children with relapsed and refractory AML [150]. A phase I trial in pediatric AML, post-HSCT relapse, showed donor-derived NK cells persisting for up to 6 months with sustained anti-leukemic activity [137]. However, adoptive transfer of NK cells did not decrease the cumulative incidence of relapse (0.393 vs. 0.35; *p* = 0.556) and did not improve event-free (60.7 ±10.9% vs. 69.1 ±  6.8%; *p* =0.553) or OS (84.2 ±8.5% vs. 79.1 ±6.6%; *p* =0.663) over chemotherapy alone [151]. The lack of benefit may result from insufficient numbers and limited persistence of alloreactive donor NK cells but does not preclude its potential usefulness during other phases of therapy, or in combination with other immunotherapeutic agents.

Another innovative approach is establishing memory-like NK cells by pre-treatment with IL-12, IL-18, and IL-15. Memory-like NK cells are highly reactive toward AML cells, both in vitro and in a clinical setting in vivo [152, 153]. In pediatric AML patients, post-HSCT relapse, adoptively transferred donor-derived memory-like NK cells were well tolerated and demonstrated anti-leukemic efficacy and safety [152, 154]. In a phase I clinical trial, clinical responses to memory-like NK cells were observed in five of nine evaluable patients, including four complete remissions [154]. Phase II clinical trials based on cytokine-induced memory-like NK cells are currently ongoing in children with relapsed/refractory AML (NCT04354025 and NCT03068819).

In adoptive NK cell transfer, the interaction between NK cell activating/inhibitory receptors and AML cells is crucial for immune response. To enhance the specificity and cytotoxicity, in adult AML, alternative NK-cell-based immunotherapies have been developed, such as CAR-modified-NK cells, monoclonal antibodies, and bi-specific killer cell engager (BiKE) or tri-specific killer cell engager (TriKE) [107]. While antibodies targeting NK cell inhibitory receptors have made great progress in adult AML, they are at the initial phase of research in pediatric AML. Notably, the CD16xCD33 BiKE demonstrated efficacy in both adult and pediatric AML, enhancing NK cell cytotoxicity, degranulation, and cytokine production against CD33+ AML [155]. Another example is the TriKE SAR443579, a trifunctional natural killer cell engager targeting the CD123 tumor antigen on cancer cells and co-engaging NKp46 and CD16a on NK cells. It showed potent antitumoral activity in preclinical studies and is being tested in both adult and pediatric patients with relapsed or refractory AML (NCT05086315) [156].

Despite being limited, cell therapy with genetically modified immune effector cells, such as CAR-T and CAR-NK, is also being explored in the clinical based on preclinical data and holds the promise to improve outcomes for pediatric and adult AML [157, 158]. There are still several challenges that have to be addressed, including finding the right antigen with low “off-tumor” toxicity or targeting multiple antigens to prevent immune escape and to overcome the immunosuppressive AML microenvironment.

Overall, exploring the application of cell of NK cell-based immunotherapies in pediatric AML holds significant potential for advancing treatment. This approach aims for greater precision, reduced side effects, and improved outcomes for young patients with AML.

## Conclusion

The characterization of the innate immune system in the TME of adult and pediatric AML is still limited. However, innate immune cells appear to play a key role in supporting leukemogenesis and immune evasion by AML, and they have been associated with resistance to standard therapies. Specifically, in AML, macrophages and MDSCs support an immunosuppressive TME, NK cells are dysfunctional, and cDCs are hardly there or absent. Therefore, targeting innate immune cells appears to be a promising strategy to overcome the immunosuppressive TME and potentially enhance the effect of T cell-based immunotherapies. Several strategies targeting myeloid cells have been developed and are currently in trials for solid and hematological malignancies, including adult AML. These include efforts aimed at decreasing the number of myeloid cells or manipulating their phenotypes. Moreover, targeting specific inhibitory receptors expressed on myeloid cells represents another promising approach. These therapies are rarely applied in clinical trials in adult AML and have not been investigated in pediatric AML yet. Alternatively, targeting NK cells can induce an anti-leukemic effect and is currently in clinical trials for both adult and pediatric AML. However, the efficacy of NK cell-based immunotherapy needs to be confirmed in large sample sizes, especially for pediatric AML.

A better understanding of the innate immune components of the TME is urgently needed to improve the ability to guide immunotherapeutic interventions in patients with AML. Moreover, it is still unknown how chemotherapy affects the number and phenotype of innate immune cells in the TME. Since immunotherapies in adult AML have mainly been applied to refractory/relapsed AML, it is important to understand the composition of the TME not only at diagnosis but also in the relapse and refractory setting. Furthermore, even if data regarding the immune cell phenotype are still limited for both adult and pediatric AML, these data suggest that results from immunotherapy trials in adult AML cannot simply be extrapolated to pediatric AML.

Overall, directing attention to the innate immune system presents a dual strategy of targeting tumor cells and the tumor microenvironment, reducing the chances of future niche-supported relapse events. Given the molecular heterogeneity of AML and the natural diversity of AML blasts, the best strategy to beat AML will eventually be to combine or sequentially apply chemotherapy, immunotherapy, and target therapy.
